# Analysis of Interleukin-8 Gene Variants Reveals Their Relative Importance as Genetic Susceptibility Factors for Chronic Periodontitis in the Han Population

**DOI:** 10.1371/journal.pone.0104436

**Published:** 2014-08-07

**Authors:** Nan Zhang, Yuehong Xu, Bo Zhang, Tianxiao Zhang, Haojie Yang, Bao Zhang, Zufei Feng, Dexing Zhong

**Affiliations:** 1 Department of Dentistry, the First Affiliated Hospital, College of Medicine, Xi'an Jiaotong University, Xi'an, China; 2 Key Laboratory of Environment and Genes Related to Diseases, College of Medicine, Xi'an Jiaotong University, Xi'an, China; 3 School of Life Science and Technology, Xi'an Jiaotong University, Xi'an, China; 4 School of Electronic and Information Engineering, Xi'an Jiaotong University, Xi'an, China; 5 Department of Psychiatry, Washington University in Saint Louis, Saint Louis, Missouri, United States of America; 6 Key Laboratory of National Ministry of Health for Forensic Sciences, College of Medicine, Xi'an Jiaotong University, Xi'an, China; 7 The Second Department of Orthopedics, the Second Affiliated Hospital, College of Medicine, Xi'an Jiaotong University, Xi'an, China; Boston University, United States of America

## Abstract

Interleukin (IL)-8, an important chemokine that regulates the inflammatory response, plays an important role in periodontitis. Previous studies indicate that certain IL-8 gene polymorphisms are associated with periodontitis susceptibility in some populations. However, the literature is somewhat contradictory, and not all IL-8 polymorphisms have been examined, particularly in Han Chinese individuals. The aim of this study was to investigate the association of every IL-8 SNP with chronic periodontitis in Han Chinese individuals. We analyzed 23 SNPs with minor allele frequency (MAF)≥0.01, which were selected from 219 SNPs in the NCBI dbSNP and preliminary HapMap data analyses from a cohort of 400 cases and 750 controls from genetically independent Han Chinese individuals. Single SNP, haplotype and gender-specific associations were performed. We found that rs4073 and rs2227307 were significantly associated with chronic periodontitis. Further haplotype analysis indicated that a haplotype block (rs4073-rs2227307-rs2227306) that spans the promoter and exon1 of IL-8 was highly associated with chronic periodontitis. Additionally, the ATC haplotype in this block was increased 1.5-fold in these cases. However, when analyzing the samples by gender, no significant gender-specific associations in IL-8 were observed, similar to the results of haplotype association analyses in female and male subgroups. Our results provide further evidence that IL-8 is associated with chronic periodontitis in Han Chinese individuals. Furthermore, our results confirm previous reports suggesting the intriguing possibilities that IL-8 plays a role in the pathogenesis of chronic periodontitis and that this gene may be involved in the etiology of this condition.

## Introduction

Oral Gram-negative bacteria trigger periodontitis, which produces an inflammatory response in a susceptible host [1]. Individual variations in the host's immune response, which are influenced by environmental and genetic characteristics, account for the predisposition, initiation and progression of periodontitis [2]. Environmental factors, such as hormones, diabetes and drugs, modify preexisting periodontal conditions [3]. Among these factors, tobacco use is one of the main risk factors for periodontitis, because tobacco affects the oral environment, gingival vasculature, inflammatory and immunological responses, as well as the healing potential of periodontal connective tissues [4]–[6]. Furthermore, increasing evidence suggests that genetic factors are important risk factors for predicting the susceptibility to chronic periodontitis [7].

Several studies have investigated the role of genes and their variants (polymorphisms) in host responses to chronic periodontitis and the progression of this disease [8]–[11]. In some situations, genetic polymorphisms may change protein's function or expression, altering innate and adaptive immunity, which might affect disease outcomes [12]. Genetic polymorphisms may also be protective against this disease [13]. Furthermore, the immune response of patients affected by periodontitis has been widely investigated, focusing on cytokine production and its association with chronic periodontitis [14]–[16]. Genetic susceptibility to chronic periodontitis has also been studied, focusing on immune system genes, such as interleukin-8 (IL-8). The IL-8 gene, which is located on chromosome 4q12-q13, encodes the IL-8 protein. This protein is the most potent chemokine studied to date, and it is responsible for inducing chemotaxis, which is the directed migration of cells to a site of inflammation [17]. This chemokine is important for regulation of the inflammatory response and for its ability to recruit and activate acute inflammatory cells. IL-8 is also mediates the activation and migration of neutrophils, which are the first line of defense against periodontopathic bacteria, which migrate from the peripheral blood into the tissues [18]. Moreover, IL-8 is produced early in the inflammatory response, and its presence persists for a long period of time [19].

While association studies provide a promising approach for studying the genetics of complex diseases, such as schizophrenia [20]–[23], identifying individual candidate genes/variants for disease risk is also important. Previous studies indicate the association between IL-8 SNPs and periodontitis compared with healthy controls. Some studies suggest that SNPs within IL-8 are associated with susceptibility to periodontitis [24]–[28], whereas other studies failed to demonstrate associations between periodontitis and IL-8 polymorphisms [29], [30]. The association between IL-8 and chronic periodontitis has not been systematically investigated in the Han Chinese population. Despite evidence of a significant association within some populations, the contribution of IL-8 to periodontitis and its potential mechanisms of action in periodontitis remain to be elucidated. Therefore, an exploration of possible association between IL-8 polymorphisms and periodontitis is necessary among genetically independent populations. To confirm the association of IL-8 with chronic periodontitis, we performed an association study for IL-8 with chronic periodontitis in Han Chinese individuals. Additionally, this study provides further information regarding the use of IL-8 polymorphisms as markers of susceptibility to periodontal disease.

## Materials and Methods

### Patients and controls

This study was designed as a case-controlled study. The study group was composed of 1150 individuals, ranging from 27–62 years of age. It took almost one year (June 2012 to April 2013) to complete sample collection. All of the subjects were unrelated Han Chinese individuals randomly selected from the Shaanxi Province, with no migration history within the previous 3 generations. Additionally, all participants were of a similar socio-economic level, which is important because there is a strong association between low socio-economic status and a higher risk of periodontal disease [31]. All enrolled individuals answered a questionnaire to obtain information regarding dental history, family history of periodontal disease, cigarette smoking habits and general health. All of the subjects were required to have at least 10 teeth, be in good general health and be free of oral soft tissue abnormalities or severe dental caries, except for the presence of chronic periodontitis. Patients who reported the following characteristics were excluded from the study: use of orthodontic appliances, chronic anti-inflammatory drugs or immunosuppressive chemotherapy, antibiotics within the previous 3 months, chronic inflammatory diseases, a history of diabetes mellitus, hepatitis, HIV infection, nephritis, bleeding or autoimmune disorders, diseases with severe commitment of the immune function, current pregnancy or breastfeeding. Other authors have previously used these exclusion criteria [32]–[35]. Because tobacco is associated with increased clinical attachment loss (CAL) and supports alveolar bone loss, it represents an important risk factor for the initiation and progression of periodontal disease [3], [36]–[38]. The individuals enrolled in this study were all nonsmokers (never smoked before).

Individuals were categorized into the control and chronic periodontitis groups.

The healthy control group did not have signs of any periodontal disease at the time of sample collection and did not have a history of previous periodontal disease as determined by a lack of sites with probing depth (PD) >3 mm and the absence of gingival recession, CAL, and bleeding on probing (BOP). The 750 blood samples from periodontally healthy Han Chinese subjects were obtained randomly, which represented the controlled population (375 males and 375 females, aged 27 to 60 years with a mean age of 50.32±8.27 years; Table 1).

**Table 1:** Demographic characteristics and clinical parameters of chronic periodontitis and controls.

	Chronic periodontitis (n = 400)	Controls (n = 750)
Gender (male/female)	200/200	375/375
Range of age (years)	28–62	27–60
Mean age (years)	50.46±9.14	50.32±8.27
PD (mm)	5.52±0.26	NA
CAL (mm)	5.58±0.45	NA
BOP (%)	48.06±13.43	NA

Patients with chronic periodontitis often presented an amount of destruction consistent with the amount of microbial deposits, presence of subgingival calculus, probable association with local predisposing factors and a slow to moderate rate of progression. All of the chronic periodontitis subjects were previously diagnosed with moderate or severe chronic periodontal disease. Diagnosis of chronic periodontitis (CP) was established clinically and by X-ray verification, according to the criteria of the American Academy of Periodontal Disease (AAP, 1999) [39], presence of chronic gingivorrhagia, bleeding on probing, clinical attachment loss, and horizontal or vertical loss of alveolar bone. In all patients, the degree of clinical attachment loss was defined using confirmed periodontal probe. Patients with probing depths greater than 5 mm, CAL greater than 4 mm, and some degree of gingival recession and tooth mobility were chosen. This clinical form is most prevalent in adults, but its occurrence may be present in younger individuals [40]. This patient group was composed of 400 subjects (200 males and 200 females, aged 28 to 62 years with a mean age of 50.46±9.14 years; Table 1) that were recruited from the inpatient and outpatient clinical services at the First Affiliated Hospital of Xi'an Jiaotong University, the second Affiliated Hospital of Xi'an Jiaotong University and the Stomatology Hospital of Xi'an Jiaotong University.

This study was approved by the Xi'an Jiaotong University Ethics Committee. All participants completed written informed consent forms. Data related to the participants are described in Table 1.

### SNP selection and genotyping

**Figure 1:**
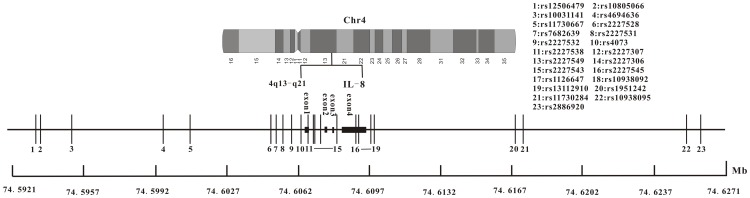
Distribution of the 23 SNPs across the IL-8 gene selected for the association analysis and their relationship with gene exons.

IL-8 polymorphisms were identified using the National Center for Biotechnology Information single nucleotide polymorphism database, and 209 SNPs were identified. For the first screen of the most common SNPs in Han Chinese chronic periodontitis patients, a MAF≥0.01 was used as the cut-off. Based on these criteria, we selected 14 SNPs in IL-8 (rs2227528, rs7682639, rs2227531, rs2227532, rs4073, rs2227538, rs2227307, rs2227549, rs2227306, rs2227543, rs2227545, rs1126647, rs10938092 and rs13112910). Next, we then searched for all SNPs with minor allele frequencies (MAF)≥0.01 between 20 kb upstream and 20 kb downstream of IL-8 in the HapMap HCB database using Haploview [41], which identified 9 SNPs (rs12506479, rs10805066, rs10031141, rs46946336, rs11730667, rs1951242, rs11730284, rs10938095 and rs2886920). Therefore, we selected 23 SNPs in the 45 kb region containing IL-8 (Fig. 1).

Patients and controls were mixed on the same plates using a double-blind procedure. Plasma samples were stored at −20°C. Genomic DNA was isolated from peripheral blood leukocytes according to the manufacturer's protocol (Genomic DNA kit, Axygen Scientific Inc., California, USA), and DNA samples were stored at −20°C for SNP analysis. SNP genotyping was performed using the Sequenom MassARRAY platform with iPLEX GOLD chemistry (Sequenom, San Diego, CA, USA), according to the manufacturer's protocol. Polymerase chain reaction (PCR) primers and locus-specific extension primers were designed using MassARRAY Assay Design software package (v. 3.1). 50 nanograms of DNA template were used in each multiplexed PCR well. PCR products were treated with shrimp alkaline phosphatase (USB, Cleveland, OH, USA) prior to use in the iPLEX GOLD primer extension reaction. Single base extension products were desalted with SpectroCLEAN resin (Sequenom), and 10 nL of the desalted product was spotted onto a 384-format SpectroCHIP using the MassARRAY Nanodispenser. Mass determination was performed with a MALDI-TOF mass spectrometer, and MassARRAY Typer 4.0 software was employed for data acquisition. The final genotype call rate of each SNP was greater than 96% and the overall genotyping call rate was 98.1%, confirming the reliability of further statistical analyses.

### Statistical analysis and power analysis

Hardy–Weinberg equilibrium (HWE) for each SNP was assessed using GENEPOP v4.0. Allelic and genotypic association tests were performed using CLUMP v2.4 with 10,000 simulations, and this program employed an empirical Monte Carlo test of significance through simulation. To control for possible confounding effects, age and gender were used as independent variables in a multiple logistic regression analysis for adjustment by commercially available software (Statistical Package for Social Sciences, version 16.0 for windows, SPSS Inc., Chicago, IL, USA). The D′ values for each pair of markers were calculated using the software program 2LD [42]. Haplotype frequencies were estimated using GENECOUNTING v2.2, which computes maximum-likelihood estimates of haplotype frequencies from unknown phase data using an expectation–maximization algorithm [43]. The significance of a haplotypic association with chronic periodontitis was evaluated using a likelihood ratio test, followed by permutation testing that compared the estimated haplotype frequencies in patients and controls [43], [44]. Differences were considered significant when the p value was less than 0.05. For haplotype analyses, the global p value was based on a comparison of the frequency distribution of all possible combinations of haplotypes among patients and controls. Furthermore, we performed power calculations for case–control genetic association analyses using PGA v2.0 [45]. Our sample size can detect SNP and haplotype associations with 91% and 85% power, respectively, at a false positive rate of 5%, and a presumed minimum odds ratio (OR) of 1.5.

## Results

In total, 23 SNPs in the 45 kb region containing IL-8 were genotyped in 400 cases and 750 controls. The allele and genotype frequencies of all in case and control SNPs, including the results of the HWE test, are shown in Table 2 and Table S1. All SNPs were highly polymorphic in both cases and controls, with the exception of 7 SNPs (rs2227528, rs7682639, rs2227531, rs2227532, rs2227538, rs2227549 and rs2227545) in IL-8, and all SNPs were in HWE in both groups. First, we conducted a single SNP association analysis. When all of the samples were considered, we observed a significant association for rs4073 (p = 0.028251; OR = 1.207; 95% CI 1.014–1.437), and rs2227307 (p = 0.003455; OR = 1.312; 95% CI 1.098–1.567). Genotype association analyses for the two SNPs suggested a similar pattern with a significant p value (p = 0.014604, p = 0.008132, respectively). Because we observed significant associations for rs4073 in the recessive model (p = 0.004) and rs2227307 in the dominant, recessive and co-dominant models (p = 0.007, 0.021 and 0.035, respectively) (Table 2), a multiple logistic regression analysis was used for rs4073 in the recessive model and used for rs2227307 in the dominant, recessive and co-dominant models to evaluate the associations of alleles or genotypes with chronic periodontitis susceptibility, while adjusting for modifying factors, such as subject age and gender, to control for possible confounding effects (Table 3). A significant association was observed between age and chronic periodontitis (p = 0.002), while gender was not associated with chronic periodontitis (Table 3). The rs4073 TT genotype and rs2227307 T allele were identified as significant risk factors for chronic periodontitis after adjustment for age and gender (OR = 1.451, 95% CI = 1.196–1.628, P = 0.017; OR = 1.597, 95% CI = 1.206–1.985, P = 0.029; respectively) (Table 3).

**Table 2:** Allele and genotype frequency of single SNP association analysis.

SNP Makers	Allele Freq. (%)		p-value^1^	Genotype Freq. (%)			p-value^1^	H-W E p value	OR^2^ 95%CI		p-value	
										Dominant	Recessive	Co-dominant
rs4073	A	T		AA	AT	TT						
Case	318(39.8)	482(60.2)	***0.028251***	71(17.7)	177(44.2)	152(38.1)	***0.014604***	0.121	1.207	0.702	***0.004***	0.547
Control	668(44.5)	833(55.5)		140(18.7)	387(51.6)	223(29.7)		0.222	(1.014–1.437)			
rs2227307	G	T		GG	GT	TT						
Case	282(35.2)	518(64.8)	***0.003455***	44(11.1)	193(48.2)	163(40.7)	***0.008132***	0.258	1.312	***0.007***	***0.021***	***0.035***
Control	623(41.5)	877(58.5)		127(16.9)	369(49.2)	254(33.9)		0.716	(1.098–1.567)			
rs2227306	C	T		CC	CT	TT						
Case	574(71.8)	226(28.2)	0.063353	209(52.3)	156(39.0)	35(8.7)	0.119011	0.460	1.195	0.538	0.063	0.977
Control	1020(68.0)	480(32.0)		344(45.9)	332(44.2)	74(9.9)		0.669	(0.990–1.443)			

OR: odds ratio; CI: confidence interval.

1 P values of the normal chi-square statistics from Monte Carlo stimulation using CLUMP (T1), and significant p values are in italic bold.

2 OR refers to risk allele odds ratio in cases and controls.

**Table 3:** Results of logistic regression analysis for susceptibility to chronic periodontitis.

Characteristics of subjects		Case	Control	OR	95%CI	p-value
Age				1.292	1.168–1.435	***0.002***
Gender	Male	200(34.78%)	375(65.22%)	Reference		
	Female	200(34.78%)	375(65.22%)	1.003	0.832–1.375	0.974
rs4073 TT carriage	Non-carriers	248(32.00%)	527(68.00%)	Reference		
	Carriers	152(40.53%)	223(59.47%)	1.451	1.196–1.628	***0.017***
rs2227307 T allele carriage	Non-carriers	44(25.73%)	127(74.27%)	Reference		
	Carriers	356(36.36%)	623(63.64%)	1.597	1.206–1.985	***0.029***
rs2227307 TT carriage	Non-carriers	237(32.33%)	496(67.67%)	Reference		
	Carriers	163(39.09%)	254(60.91%)	1.328	0.863–1.794	0.081
rs2227307 GT carriage	Non-carriers	207(35.20%)	381(64.80%)	Reference		
	Carriers	193(34.34%)	369(65.66%)	0.959	0.721–1.288	0.163

OR: odds ratio; CI: confidence interval.

Significant p values are in italic bold.

Table 4 presents the results of LD tests (noted as D′ and r^2^) between pairs of SNP markers within IL-8 for the respective control groups. According to these results, LD (D′>0.8) was observed in the five-SNP linkage disequilibrium estimation. When combining the allele frequency data with the LD, the associated SNPs, rs4073 and rs2227307, were detected in the same LD block as rs2227306 (D′>0.8 between them, Table 4). Next, we performed the haplotypic association analysis of the LD block mentioned above (Table 5). Tests of the common three-marker haplotype (frequency >0.025) association for rs4073, rs2227307, and rs2227306 indicated a significant association between these SNPs and chronic periodontitis (p<0.001, global permutation). Some haplotypes of these three SNPs were significantly associated with chronic periodontitis. For example, HAP2 (ATC) was significantly associated with chronic periodontitis, and its frequency increased nearly 1.5-fold in cases (corrected p = 0.039). Due to higher frequencies in the controls, HAP3 (AGT) may provide a protective effect with nearly a 1.8-fold prevalence in controls (corrected p<0.001) (Table 5).

**Table 4:** Estimation of LD between each pair of loci within IL-8.

	rs4073	rs2227307	rs2227306	rs2227543	rs1126647
rs4073	-	0.743	0.322	0.028	0.015
rs2227307	0.916	-	0.321	0.039	0.022
rs2227306	0.924	0.981	-	0.097	0.098
rs2227543	0.257	0.322	0.331	-	0.099
rs1126647	0.222	0.284	0.347	0.369	-

D′-value are shown below the subtraction sign, and r^2^-value are shown above the subtraction sign.

**Table 5:** Haplotypes frequency and association analysis.

Haplotype				Genecounting (frequency %)				
ID	SNP6	SNP7	SNP8	Case	Control	p-value^1^	correction^2^	Global p^3^
HAP1	T	T	C	40.3	39.4	0.692		***<0.001***
*HAP2* 4	A	T	C	20.3	15.6	***0.005***	***0.039***	
*HAP3* 4	A	G	T	10.6	18.2	***<0.001***	***<0.001***	
HAP4	T	G	T	13.4	10.3	***0.028***	0.227	
HAP5	A	G	C	7.02	8.75	0.147		
HAP6	T	G	C	4.15	4.28	0.872		

Significant p-values are in italic bold. Common Haplotypes are shown, if frequency more than 2.5%.

1 Based on 10000 permutations.

2 Corrected by Bonferroni.

3 Based on comparison of frequency distribution of all haplotypes for the combination of SNPs.

4 Haplotypes in italics are the significant ones in the study.

To examine whether gender would play a key role in the association, we analyzed our data by separating females and males according to the above results. Neither rs4073 nor rs2227307 displayed gender-specific associations with chronic periodontitis (Table 6). Moreover, haplotype association analyses were similar to the single-SNP analysis results, revealing no gender-specific association in females or males (Table 7).

**Table 6:** Allele and genotype association analysis in female and male.

Marks		Allele frequency (%)		p value^1^	Genotype frequency (%)			H-W E p value	p value^1^	OR^2^, 95%CI	p value		
											Dominant	Recessive	Co-dominant
rs4073		A	T		AA	AT	TT						
Female	Case	157(39.3)	243(60.7)	0.110589	34(17.1)	89(44.4)	77(38.5)	0.327	0.103541	1.223	0.735	0.066	0.711
	Control	331(44.1)	419(55.9)		68(18.1)	195(52.0)	112(29.9)	0.290		(0.955–1.565)			
Male	Case	161(40.3)	239(59.7)	0.126923	37(18.3)	88(44.0)	75(37.7)	0.226	0.139648	1.192	0.838	0.054	0.633
	Control	337(44.9)	413(55.1)		72(19.3)	192(51.2)	111(29.5)	0.501		(0.932–1.525)			
rs2227307		G	T		GG	GT	TT						
Female	Case	142(35.5)	258(64.5)	***0.035592***	25(12.5)	92(46.0)	83(41.5)	0.950	0.111624	1.309	***0.045***	0.881	0.238
	Control	314(41.9)	436(58.1)		67(17.9)	180(48.0)	128(34.1)	0.784		(1.018–1.682)			
Male	Case	140(34.9)	260(65.1)	***0.044555***	19(9.7)	101(50.4)	80(39.9)	0.123	0.063942	1.316	***0.031***	0.127	0.070
	Control	308(41.1)	442(58.9)		60(15.9)	189(50.4)	126(33.7)	0.427		(1.023–1.693)			
rs2227306		C	T		CC	CT	TT						
Female	Case	290(72.5)	110(27.5)	0.191898	105(52.5)	80(40.0)	15(7.5)	0.965	0.398158	1.196	0.522	0.183	0.794
	Control	516(68.8)	234(31.2)		175(46.7)	166(44.2)	34(9.1)	0.567		(0.914–1.564)			
Male	Case	284(71.1)	116(28.9)	0.186387	104(52.1)	76(38.0)	20(9.9)	0.287	0.273542	1.195	0.803	0.113	0.774
	Control	504(67.2)	246(32.8)		169 (45.1)	166(44.2)	40(10.7)	0.959		(0.917–1.557)			

OR: odds ratio; CI: confidence interval.

Significant p values are in italic bold. CI: confidence interval; OR: odds ratio.

1 P values of the normal chi-square statistics from Monte Carlo stimulation using CLUMP (T1), and significant p values are in italic bold.

2 OR refers to risk allele odds ratio in cases and controls.

**Table 7:** Haplotypes frequency and association analysis in female and male.

Haplotype	Female		Male		p value^1^		
Frequency (%)	Case	Control	Case	Control	Female	Male	All
rs4073- rs2227307- rs2227306			Global-p value***<0.001***				
ATC	21.2	16.4	19.4	14.8	***0.042***	***0.041***	***0.039***
AGT	11.3	18.1	9.65	18.5	***<0.001***	***<0.001***	***<0.001***

1 Based on 10000 permutations, and significant p values are in italic bold.

## Discussion

IL-8 is a chemokine related to the initiation and amplification of acute inflammatory responses and the chronic inflammatory process [46]. The purpose of this study was to explore the relationship between all SNPs within the IL-8 gene and chronic periodontitis in Han Chinese individuals. In this study, we present evidence for the association of markers that are mapped to the 45 kb region of IL-8 gene with chronic periodontitis. We identified a significant association in the region between rs4073 and rs2227306, and several lines of evidence suggest that the observed association is unlikely to be an artifact. First, both the single SNP and the haplotype-based association analyses support the association. Second, population stratification is an unlikely explanation because all of our samples are from the same geographical region. Lastly, similar results were obtained from other genetically independent populations in previous studies [25], [26], [28], [47], reaffirming the observed association.

In association studies, it is critical to identify common risk variants in different populations. To examine whether common risk variants exist in genetically independent populations, we compared our results with those of previous studies. Individual differences in interleukins levels can be attributed to gene polymorphisms, especially when these polymorphisms are located within exons or promoters. The common rs4073 A/T polymorphism in the IL-8 promoter influences the production and expression of IL-8. The rs4073 A allele increases IL-8 production both in vitro and in vivo [48], [49]. Lee et al. [49] reported that the presence of the rs4073 T allele of IL-8 exerts a 2–5-fold higher transcriptional activity than the rs4073 A allele. Two additional studies evaluated the association of the IL-8 rs4073 A/T polymorphism and periodontitis, but contradictory results were obtained [25], [50]. Kim et al. [29] found no association between the genotype distribution and allele frequency of this gene and chronic periodontitis in the Brazilian population. In contrast, Andia et al. [25] discovered a significant association between the IL-8 rs4073 A/T polymorphism and chronic periodontitis in Brazilian nonsmokers, with a higher frequency of the A allele in the disease group compared to the control group. The results of our study do not agree with these studies. We found that the A allele of rs4073 displayed a tendency to be lower in cases compared to controls in the Han Chinese population. Collectively, the consistency between these studies in different ethnic populations provides strong evidence that the rs4073 polymorphism in the IL-8 gene may be involved in chronic periodontitis susceptibility. Additionally, we also observed other differences among these studies. Rs2227307 had a significant allelic and genotypic association with chronic periodontitis in our analysis; however, only genotypic association data were similar to that reported by Scarel-Caminaga et al. [28]. To evaluate potential confounding effects that could cause bias in this association study of chronic periodontitis, important factors known to influence the pathogenesis of chronic periodontitis were assessed by multiple logistic regression analysis. Multiple logistic regression analysis revealed that age was associated with chronic periodontitis (Table 3). Regarding age, an important risk factor for periodontitis, Grossi et al. (1995) observed an OR = 2.6 (95% CI: 1.75–3.83) for the age group 35–44 years and an OR = 24.08 (95% CI: 15.93–36.29) for the age group 65–74 years, which are similar to the results of our study (Table 3) [51]. There is evidence that both the prevalence and severity of chronic periodontitis increase with increasing age [52]. However, the age effect could conceivably represent the cumulative effect of prolonged exposure to other risk factors [53].

The ability to draw conclusions regarding associations based on the analysis of individual SNPs is limited [54]. Therefore, to obtain stronger statistical evidence, we performed a haplotype analysis. Haplotype analysis uses additional information regarding the linkage between typed markers. The results of haplotype frequency estimation for three-SNP combinations (rs4073-rs2227307-rs2227306) indicated significant associations with chronic periodontitis (p<0.001, global permutation). The frequency of the ATC haplotype was 20.3% in cases and 15.6% in controls, demonstrating that individuals with ATC were almost 1.5 times more likely to develop chronic periodontitis than individuals carrying other haplotypes. This result was consistent with that reported by Scarel-Caminaga et al. [28]. However, a protective haplotype, AGT, which was almost 1.8 times more prevalent in controls than in cases, displayed a significant positive association with chronic periodontitis in our studies. Additionally, no gender-specific associations were observed in single SNP or haplotype analyses.

Genetic backgrounds vary among ethnic populations; therefore, differences in the results among these studies might be the result of ethnic differences and the genetic heterogeneity that existing in the IL-8 gene, despite some similarities in general association patterns. Nevertheless, our results should be validated in other populations or with a larger sample size in this population. This validation is required because the statistically significant results could occur by chance, and because the associations were not significant after an adjustment for multiple testing, despite remaining significant after 10,000 permutation tests. In addition, SNPs and haplotype structures can vary amongst different ethnic groups. Therefore, our data should be interpreted with caution, as the combination of these polymorphisms with others in different genes may also be important to define the role of these polymorphisms in the pathogenesis of chronic periodontitis.

A major limitation of the current study was that we did not perform further replication analyses for the possible risk of the SNPs identified in the study due to the lack of another cohort of patients and controls. However, as a replication study to confirm the previous studies and providing further evidence for the association of IL-8 with chronic periodontitis, our study did not appear extremely heterogeneous, and it could be considered reasonable and reliable. Additionally, although we selected subjects with no migration history within the previous three generations to control population stratification caused by genetic factors, we did not focus on other possible factors leading to population stratification; thus, we cannot completely rule out hidden stratification interference. Therefore, our findings should be considered preliminary. Given that the pathomechanism of chronic periodontitis includes further cytokines, chemokines, and pattern-associated molecules, additional follow-up studies are required to find possible causal variants. Ideally, these studies will involve the use of more sophisticated techniques of genetic investigation, such as DNA microarrays, to better understand the involvement of genetic factors in chronic periodontitis. Additionally, it is important to investigate the interaction of host genetics with clinical parameters and the immune response because chronic periodontitis is a complex disease, and the interaction of different factors has been insufficiently evaluated.

In recent years, particular interest has been given to investigating functional polymorphisms in candidate genes for disease association. The expression levels of a protein may be modulated by genetic polymorphisms in regulatory regions of the gene, mainly the promoter region [55]. Considering that some IL-8 polymorphisms were previously reported to be associated with higher IL-8 production and that a significantly higher level of this protein was found in the gingival crevicular fluid from patients with periodontitis [56], we hypothesize that the genetic differences between individuals in relation to IL-8 production may somehow predispose them to periodontal disease. The significantly associated SNPs identified in our study are not randomly distributed over the gene. Rather, these SNPs in the promoter and intron 1 are in the same LD block spanning the promoter and exon 1 of IL-8. Therefore, the significant associations in our study may be of interest for future studies. First, there is a cluster of significantly associated SNPs that span the promoter and exon 1 of IL-8, indicating a region of interest that might harbor functionally relevant variants. Second, we provide additional data in agreement with the previously reported IL-8 polymorphisms that are associated with chronic periodontitis susceptibility. To remedy the mentioned shortcomings, we will try to observe the effects of these polymorphisms on periodontal systemic therapy in future studies.

In summary, our work provides supportive evidence for the association of IL-8 with chronic periodontitis. To our knowledge, this is the first study to demonstrate all SNPs between 20 kb upstream and 20 kb downstream of the IL-8 gene with chronic periodontitis in the Han Chinese population. Moreover, as an intriguing new insight into the pathogenesis of chronic periodontitis, we also confirmed previous reports suggesting that this gene plays an important role in the etiology of this condition. Because chronic periodontitis is multifactorial in nature, involving interactions between genes, the environment and lifestyle, genetic periodontal risk assessments may be valuable in developing preventive, diagnostic and therapeutic strategies against the incidence and progression of this condition. Given the complex patterns of findings from association studies focusing on chronic periodontitis and its underlying genetic heterogeneity, further inquiries and wider replications are required, particularly within different ethnic samples.

## Supporting Information

Table S1 **Allele and genotype frequency of other 13 SNPs association analyses.**
(DOC)
