# Supplementary material for: Analysis of Interleukin-8 Gene Variants Reveals Their Relative Importance as Genetic Susceptibility Factors for Chronic Periodontitis in the Han Population
Source: PLoS One. 2014 Aug 7;9(8):e104436. doi: 10.1371/journal.pone.0104436 (PMC4125212; doi:10.1371/journal.pone.0104436)
Supplement: Table S1 — Allele and genotype frequency of other 13 SNPs association analyses. (DOC) [file pone.0104436.s001.doc]

Table S1 Allele and genotype frequency of other 13 SNPs association analyses

| Makers | | Allele Freq. (%) | | p-value^1^ | Genotype Freq. (%) | | | p-value^1^ | H-W E p value | OR^2^  95%CI |
| --- | --- | --- | --- | --- | --- | --- | --- | --- | --- | --- |
| ID | bp |  | |  |  | | |  |  |  |
| rs12506479 | 74,592,1 | C | T |  | CC | CT | TT |  |  |  |
| Case | 61 | 47.2 | 52.8 | 0.664380 | 21.8 | 50.8 | 27.4 | 0.902390 | 0.701 | 1.032 |
| Control |  | 46.3 | 53.7 |  | 21.4 | 49.8 | 28.8 |  | 0.968 | (0.869-1.226) |
| rs10805066 | 74,592,3 | C | G |  | CC | CG | GG |  |  |  |
| Case | 90 | 78.3 | 21.7 | 0.269753 | 60.4 | 35.8 | 3.8 | 0.224186 | 0.285 | 1.118 |
| Control |  | 80.2 | 19.8 |  | 64.8 | 30.8 | 4.4 |  | 0.408 | (0.905-1.380) |
| rs10031141 | 74,594,0 | A | G |  | AA | AG | GG |  |  |  |
| Case | 43 | 62.6 | 37.4 | 0.297079 | 38.4 | 48.4 | 13.2 | 0.478958 | 0.501 | 1.098 |
| Control |  | 60.4 | 39.6 |  | 36.6 | 47.6 | 15.8 |  | 0.892 | (0.920-1.310) |
| rs4694636 | 74,598,8 | G | T |  | GG | GT | TT |  |  |  |
| Case | 09 | 38.3 | 61.7 | 0.234348 | 13.5 | 49.6 | 36.9 | 0.316767 | 0.323 | 1.113 |
| Control |  | 40.8 | 59.2 |  | 16.9 | 47.8 | 35.3 |  | 0.774 | (0.934-1.327) |
| rs11730667 | 74,600,2 | A | G |  | AA | AG | GG |  |  |  |
| Case | 11 | 66.5 | 33.5 | 0.284385 | 44.3 | 44.4 | 11.3 | 0.544558 | 0.945 | 1.103 |
| Control |  | 68.7 | 31.3 |  | 46.8 | 43.8 | 9.4 |  | 0.613 | (0.918-1.324) |
| rs2227543 | 74,607,9 | C | T |  | CC | CT | TT |  |  |  |
| Case | 10 | 63.1 | 36.9 | 0.277217 | 38.5 | 49.2 | 12.3 | 0.207344 | 0.258 | 1.103 |
| Control |  | 65.4 | 34.6 |  | 43.4 | 44.0 | 12.6 |  | 0.447 | (0.923-1.319) |
| rs1126647 | 74,609,0 | A | T |  | AA | AT | TT |  |  |  |
| Case | 45 | 70.9 | 29.1 | 0.501514 | 50.4 | 41.0 | 8.6 | 0.745439 | 0.898 | 1.061 |
| Control |  | 72.2 | 27.8 |  | 52.8 | 38.8 | 8.4 |  | 0.359 | (0.877-1.283) |
| rs10938092 | 74,609,7 | A | G |  | AA | AG | GG |  |  |  |
| Case | 15 | 34.8 | 65.2 | 0.938839 | 11.2 | 47.2 | 41.6 | 0.796020 | 0.422 | 1.003 |
| Control |  | 34.9 | 65.1 |  | 12.2 | 45.4 | 42.4 |  | 0.981 | (0.838-1.201) |
| rs13112910 | 74,609,7 | A | G |  | AA | AG | GG |  |  |  |
| Case | 55 | 40.6 | 59.4 | 0.585836 | 15.9 | 49.4 | 34.7 | 0.812188 | 0.628 | 1.049 |
| Control |  | 41.8 | 58.2 |  | 17.4 | 48.8 | 33.8 |  | 0.935 |  |
| rs1951242 | 74,617,2 | C | T |  | CC | CT | TT |  |  | (0.881-1.249) |
| Case | 82 | 38.8 | 61.2 | 0.588412 | 15.0 | 47.6 | 37.4 | 0.770239 | 0.964 | 1.049 |
| Control |  | 37.6 | 62.4 |  | 13.4 | 48.4 | 38.2 |  | 0.389 | (0.879-1.252) |
| rs11730284 | 74,617,6 | A | C |  | AA | AC | CC |  |  |  |
| Case | 45 | 65.6 | 34.4 | 0.569941 | 43.4 | 44.4 | 12.2 | 0.828140 | 0.745 | 1.055 |
| Control |  | 66.8 | 33.2 |  | 45.3 | 43.0 | 11.7 |  | 0.403 | (0.880-1.264) |
| rs10938095 | 74,626,1 | C | T |  | CC | CT | TT |  |  |  |
| Case | 61 | 34.1 | 65.9 | 0.520662 | 10.5 | 47.2 | 42.3 | 0.420119 | 0.315 | 1.061 |
| Control |  | 32.8 | 67.2 |  | 11.2 | 43.2 | 45.6 |  | 0.583 | (0.885-1.273) |
| rs2886920 | 74,626,9 | C | T |  | CC | CT | TT |  |  |  |
| Case | 45 | 66.1 | 33.9 | 0.379864 | 43.0 | 46.2 | 10.8 | 0.614177 | 0.537 | 1.082 |
| Control |  | 64.3 | 35.7 |  | 41.3 | 46.0 | 12.7 |  | 0.957 | (0.903-1.296) |

CI: confidence interval; OR: odds ratio

1. p values of the normal chi-square statistics from Monte Carlo stimulation using CLUMP (T1).

2. OR refers to risk allele odds ratio in cases and controls.
